# Food choices and practices during pregnancy of immigrant and Aboriginal women in Canada: a study protocol

**DOI:** 10.1186/1471-2393-11-100

**Published:** 2011-12-07

**Authors:** Gina MA Higginbottom, Helen Vallianatos, Joan Forgeron, Donna Gibbons, Rebecca Malhi, Fabiana Mamede

**Affiliations:** 1 Faculty of Nursing, University of Alberta, Edmonton, AB, Canada; 2 Department of Anthropology, Faculty of Arts, Edmonton, AB, Canada; 3 Lois Hole Hospital for Women, Alberta Health Services, Edmonton, AB, Canada; 4 Department of Maternal-Infant and Public Health Nursing, University of São Paulo at Ribeirão Preto College of Nursing, Brasil

## Abstract

**Background:**

Facilitating the provision of appropriate health care for immigrant and Aboriginal populations in Canada is critical for maximizing health potential and well-being. Numerous reports describe heightened risks of poor maternal and birth outcomes for immigrant and Aboriginal women. Many of these outcomes may relate to food consumption/practices and thus may be obviated through provision of resources which suit the women's ethnocultural preferences. This project aims to understand ethnocultural food and health practices of Aboriginal and immigrant women, and how these intersect with respect to the legacy of Aboriginal colonialism and to the social contexts of cultural adaptation and adjustment of immigrants. The findings will inform the development of visual tools for health promotion by practitioners.

**Methods/Design:**

This four-phase study employs a case study design allowing for multiple means of data collection and different units of analysis. Phase 1 consists of a scoping review of the literature. Phases 2 and 3 incorporate pictorial representations of food choices (photovoice in Phase 2) with semi-structured photo-elicited interviews (in Phase 3). The findings from Phases 1-3 and consultations with key stakeholders will generate key understandings for Phase 4, the production of culturally appropriate visual tools. For the scoping review, an emerging methodological framework will be utilized in addition to systematic review guidelines. A research librarian will assist with the search strategy and retrieval of literature. For Phases 2 and 3, recruitment of 20-24 women will be facilitated by team member affiliations at perinatal clinics in one of the city's most diverse neighbourhoods. The interviews will reveal culturally normative practices surrounding maternal food choices and consumption, including how women negotiate these practices within their own worldview and experiences. A structured and comprehensive integrated knowledge translation plan has been formulated.

**Discussion:**

The findings of this study will provide practitioners with an understanding of the cultural differences that affect women's dietary choices during maternity. We expect that the developed resources will be of immediate use within the women's units and will enhance counseling efforts. Wide dissemination of outputs may have a greater long term impact in the primary and secondary prevention of these high risk conditions.

## Background

Immigrant [1] and Aboriginal [2] women may be regarded as vulnerable populations since challenges exist with respect to access and navigation of health services and more specifically maternity care services. Difficulties may be encountered in terms of accessing culturally appropriate care in addition to other challenges such as language barriers and discriminatory policy and practices. Without *culturally appropriate *health care delivery a negative trajectory of events may occur that range from simple miscommunication to life-threatening incidents [1,3]. The danger is especially severe during the perinatal period, which is for women and their children a vulnerable life stage and a sensitive period of interaction with the Canadian health care system [4]. Public health initiatives increasingly require healthcare organizations to promote, protect and contribute to reducing health inequities.

### Diversity and Health of Immigrants and Aboriginal Peoples in Canada

For this research, we utilize the Canadian Council of Refugees definition of an immigrant: *a person who has settled permanently in another country*. Immigrant women are a tremendously diverse group that includes economic skilled workers, refugees and asylum seekers, temporary foreign workers, and those without legal status [5]. Canada as a multicultural society has a long standing history of embracing diverse immigrant groups because of the strategy of utilizing immigration as a means of population expansion and nation building [6]. While the majority of immigrants during the first 70 years of the 20th century were of European origin, over the past 30 years there has been a shift with larger groups of immigrants arriving from Asia and the Middle East, the Caribbean and Central America, Africa, Oceania and other countries [7]. A recent report from Statistics Canada predicts that members of visible minority groups will comprise between 29% and 32% of Canada's population in 2031 [8].

As evidenced by the *healthy immigrant effect *[9,10] relatively healthy immigrants enter Canada, yet within 10 years a convergence is observed in terms of health status moving towards the Canadian average. A number of explanations are postulated for this, including health selection[9], acculturation and the stress of relocation that may erode health advantage [10], and distrust of Western medicine with a preference for seeking out traditional health care providers. It is important to note that the healthy immigrant effect largely affects those communities whose immigration is planned, since populations who relocate as refugees or asylum seekers are found to have compromised health status, with women often having been traumatized by war, rape and the transgression of their human rights [11]. Many may have lived in refugee camps for several years prior to immigration; therefore the pre-migration period may exert a powerful influence on health status including those related to maternal health and maternity service provision.

Aboriginal peoples is a collective name for all original peoples of Canada and their descendants, and the Constitution Act of 1982 specifies that the Aboriginal peoples in Canada consist of three groups: First Nations, Inuit and Métis [12]. Aboriginal peoples are the fastest growing population in Canada. Between 1996 and 2006, the population grew by 45 percent, almost six times faster than the non-Aboriginal population growth rate of 8 percent [13]. Aboriginal peoples in Canada are a vastly diverse population. For instance, Canada's First Nations peoples consist of more than 600 different communities, under approximately 50 different linguistically and culturally distinct groups [14]. The effects of colonization and policies associated with the Indian Act have had enormous consequences on Aboriginal peoples' health and well-being that continues to this day.

Numerous health inequities exist for Aboriginal people, which are directly and indirectly related to the social, economic, cultural, and political factors that result in a disproportionate burden of ill health at both the individual and the community level [15,16]. These health challenges and their determinants have largely been maintained by an intergenerational cycle of cumulative effects/trauma (often leading to 'normalized' patterns of ill-health and abuse) which helps explain how a disproportionate number of Aboriginal people experience social and health challenges compared to the general population [17].

### Maternal and Birth Outcomes of Immigrant and Aboriginal Women: Relevance of Food Choices and Practices

Largely epidemiological research from Canada and elsewhere has reported equal or more favourable birth outcomes for migrants [18-21] supporting an "epidemiological paradox" associated with the concept of the "healthy migrant effect". Numerous other reports highlight serious problems of equity in perinatal health outcomes [22-24] particularly for refugees [25] and other immigrants after increased lengths of stay (with the accompanying acculturation) [26,27]. A recent Canadian study found higher rates of low birthweight and full-term low birthweight (i.e., small for gestational age or SGA) for infants born to recent immigrant women [23] and immigrants living throughout Europe have been reported to be at substantial risk for pre-term delivery (24%), for perinatal mortality (50%), and for congenital malformations (61%) [22]. The hospital costs for preterm and SGA newborns are higher than those for their normal-growth counterparts by 9 and 2 times, respectively [28].

Aboriginal people experience a disproportionate burden of ill-health compared with the rest of the Canadian population [16,29] including such indices such as maternal and infant mortality [30,31]. Poorer birth outcomes including stillbirths, low birthweight infants and prematurity are often reported [32]. There is conflicting evidence to support *Aboriginality *itself to be the predisposing factor, multiple issues related to socioeconomic status in addition to medical and prenatal care also likely play a role [33].

While considerations of infant mortality and birth weight are important, these outcomes are not adequate or all-encompassing measures for evaluating maternal and infant health and well-being over their lifetimes. Aboriginal women have a greater chance to have gestational diabetes with the risk of giving birth to high birth-weight babies (> 4 kg, also known as macrosomia) [34,35]. Negative maternal characteristics and birth outcomes of immigrants include significantly higher rates of gestational diabetes (predisposing the mothers to preeclampsia and type 2 diabetes and their offspring to obesity and type 2 diabetes) [36]; dieting with low maternal weight gain (compromising both newborn and maternal health) [26]; genetic anomalies such as neural tube defects due to lack of folic acid intake [4]; and maternal anemia (increasing the risk of preterm delivery) [37]. All of these outcomes relate to food choices and practices.

Indeed investigators have documented changes in the everyday diet of immigrant women towards more processed foods and animal proteins as well as foods high in fat, salt, or sugar [38,39]. Evidence suggests an increasing prevalence of obesity post-migration because of the adoption of a Western diet [40] likely due to "obesogenic" food environments in the vicinities of many immigrants residences [41]. Conversely, immigrant women may instead internalize the dominant body norms of their settlement home (i.e., the skinny model or actress common in media images in North America, Australia, and Europe) and reduce their dietary intake, even during reproduction, to maintain or quickly return to these hegemonic body ideals [42]. The first author currently leads a project investigating maternity care experiences of immigrant and minority women which involves interviews with immigrant women, policy-makers, immigrant support agency representatives, and health care professionals in the cities of Edmonton and Brooks, representing both urban and rural regions of the province of Alberta, Canada. The analysis of data elicited in Edmonton has revealed two topics of significance for the maternal health of Canadian marginalized women: nutrition during the perinatal period, and psychological health and well-being in the post-natal period (unpublished data).

Aboriginal women living off-reserve in Canada are also likely affected by the urban obesogenic food environments. Relying on data from the 2004 Canadian Community Health Survey, a greater proportion of the diets of Aboriginal women living off-reserve, compared to non-Aboriginal women, consisted of snack foods including soft drinks (considered Other Foods by the Canada Food Guide to Healthy Eating of 1992) [43].

Successfully providing appropriate prenatal nutritional and diet education requires the legitimization and incorporation of the pervasive traditional beliefs and practices of immigrant women, which they often adhere to despite their new milieu [44,45]. Moreover, research and care related to pregnancy and parenthood for Aboriginal women needs to be based on the priorities and experiences of Aboriginal women and "turning around" the intergenerational impacts of residential schooling and colonialization [46].

### Study Aim

Maternity care nurses may face considerable challenges in conveying information to immigrant and Aboriginal women regarding the optimum food choices for pregnancy because of language difficulties and ethnocultural differences in reproductive health and food practices. Research is needed to elicit understanding of ethnocultural food choices and practices and to improve culturally based competency of maternity care. The development of nutrition education materials tailored to immigrant and Aboriginal populations and the provision of related education targeted to maternal care providers together have great potential to positively impact maternal and childhood health and well-being; both the transmission of appropriate messages on nutrition and the enhancement of accessibility and acceptability of maternal health care will contribute to these positive effects.

The aim of this project is to understand ethnocultural food and health practices and how these intersect in a particular social context of cultural adaptation and adjustment. An end product of this research will be a visual tool for each of three participating ethnocultural groups (Aboriginal, Chinese and Sudanese), that maternity nurses can use during health promotion interactions to convey the elements of an optimal nutritional diet. The tools will be graphic and pictorial and will enable women with limited English language skills or literacy levels to elicit pertinent information. Our ultimate goal is to improve the care-giving capacities of health practitioners (particularly maternity nurses in this study) working in a multicultural perinatal clinic setting. Our research question is How do health beliefs and practices during reproduction (i.e. preconception, during pregnancy, labour and birth, and postnatal period) of immigrant and Aboriginal women affect their food choices and subsequent health status?

## Methods/Design

### Methodology

We will employ a case study design incorporating a participatory approach [47]. Case study research excels at increasing understanding of complex, multivariate issues and emphasizes detailed contextual analysis of a limited number of events or conditions and their relationships. Social scientists make wide use of case study methods to examine complex, real-life situations [47]. Case studies allow for multiple means of data collection, data collection in different settings, and different units of analysis. The multiple sources of evidence used will be structured into four phases and include:

**Phase 1 - **A scoping review of the literature

**Phase 2 - **Pictorial representations of food choices (photovoice/photo-elicitation)

**Phase 3 **- Semi-structured photo-assisted narrative interviews

**Phase 4 - **Production of a culturally appropriate visual tool for each ethnocultural group (1-Aboriginal, 1-Sudanese, 1-Chinese)

The case study will be both descriptive and explanatory in nature [47]. Our endeavour will acknowledge the complexity of everyday lives and acknowledge the existence of multiple realities. Decisions regarding health and illness prevention are fraught with ambiguity as individuals strive to maintain culture and traditions whilst integrating evolving modernities and the influence of globalization in daily existence. The pervasive primacy of "Western" lifestyles may not engender the achievement of maximum health potential in other countries and/or cultures. Food practices are an important component of marking individual and group identity. How an individual reproduces or resists normative practices is a means of understanding their social location. Migration, whether between countries or within Canada (for instance movement to/from reserves) provides both opportunities and disjunctions for maternal health practices, including food practices. There is no question that social determinants affect approaches to meeting the human need for nutrients, but this research will provide a greater depth of understanding as to why and how such food practices develop.

### Phase 1 - Scoping Review of the Literature

Scoping reviews (or studies) can be defined as "exploratory projects that systematically map the literature available on a topic, identifying the key concepts, theories, sources of evidence, and gaps in the research" [48]. Scoping reviews are particularly relevant for emerging areas of research, where there is a paucity of high quality literature available to perform a full systematic review, and where the question(s) to address go beyond those related to intervention studies [49]. They can incorporate both published and grey literature covering a wide range of study designs. Moreover and of key relevance to this study, they can complement the findings from empirical research studies particularly with respect to summarizing, contextualizing, and disseminating research findings. A framework for performing scoping reviews was published by Arksey and O'Malley in 2007 [50] and the methodology has since been clarified and enhanced by Levac, Colquhoun and O'Brien [49], largely with respect to its iterative nature. The five-stage enhanced framework includes: identifying the research question (can be broad but must specify target population and concept); identifying relevant studies (using research questions and suitable team with content and methodological expertise); study selection (iterative process involving independent review and team reflection); charting the data (iterative with collective team input and "pilot" run with 5-10 studies); and collating, summarizing, and reporting the results (using three steps of analysis -using descriptive and qualitative thematic, reporting the results, and considering the meaning and implications) [49].

The scoping review for this study will have the following two research questions:

1. What is known from existing literature about immigrant and Aboriginal women's food choices and practices, including relevant ethnocultural and health beliefs, during pregnancy, delivery and the postpartum period?

2. What is known about the effects on health outcomes of immigrant and Aboriginal women and their offspring related to food choices and practices during maternity?

In addition to the above described framework, the review will draw upon established systematic review methodologies as outlined by the Centre for Reviews and Dissemination [51]. A transparent and replicable search strategy will be developed with assistance from a research librarian. A wide variety of sources will be searched, including key electronic bibliographic databases covering culture and ethnic group literature (CINAHL, MEDline, PUBmed, Cochrane Library, HAPY, ArticleFirst and JStore). In addition, websites from the publishers of key journals (Midwifery, Appetite, Food and Foodways, BMC Pregnancy and Childbirth, Journal of Transcultural Nursing, Journal of Migration and Ethnic Studies, J Nutr Educ Behaviour, Can J Public Health, and Food, Culture and Society) will be hand-searched. The reference lists of key papers will be checked for additional references, and citation searches will be conducted on key papers and authors. Correspondence letters, editorials and commentaries will be excluded. The emphasis will be on Canadian literature, however if significant international papers are identified these will be included. Grey literature will be identified mainly from reference lists of selected studies and websites of major regional, provincial, and federal health and immigration agencies. The search limits for date and language will be 1995-2010 and English.

For this review that aims to map out the state of knowledge in this field, it will be inappropriate to apply rigid inclusion criteria or use a traditional hierarchy of evidence. Instead each paper will be assessed against its ability to help answer the research questions. Two of the team members (RB, FM) will, under the supervision of the team lead (GH), independently screen and select the literature. Although not typical for scoping reviews, the methodological quality of the research papers will be assessed using established critical appraisal checklists, such as those produced by the Critical Appraisal Skills Program (CASP) [52]. Key data from the research studies meeting the inclusion criteria will be extracted into tables using a similar process to that laid out in the Centers for Research and Dissemination [51]. Two authors (FM & GH) will review another's (RB) data extraction for accuracy. Retrieved data will be stored in REFWORKS which is suitable for use by a team of researchers, as a group access code can be created. The report from this review will form an early output of the research in the form of a publication in a high quality peer-reviewed international research journal.

### Phases 2 and 3 - Photovoice and Photo-Assisted Interviews

#### Study setting

In 2006, Edmonton's population totaled 730,372, with 189,775 people identifying themselves as being foreign born [53]. The visible minority group totaled 22.9% largely being of Chinese, South Asian, Black and Filipino origin. Moreover, Edmonton holds the second largest urban Aboriginal population in Canada with a total number of 38,170 Aboriginal people living within the city. The hospital under consideration for this study is a tertiary care teaching hospital located in one of Edmonton's most diverse neighbourhoods. The region is home to many immigrant newcomers (with large contributions from Chinese and Sudanese populations) and older European immigrants from Italy, Poland and the Ukraine; as well it is the hub for numerous businesses with owners of an ethnic origin. Therefore, the population diversity of this area mirrors or exceeds the general population of Edmonton. The Maternal Fetal Medicine and Obstetric Medicine (Perinatal) Clinics at the Royal Alexandra Hospital's (RAH) Lois Hole Hospital for Women (LHHW) provide consultation services for preconceptual counselling, prenatal screening, diagnosis, and treatment for women who are experiencing high risk conditions in pregnancy. Many of the high risk conditions, including obesity, diabetes, hypertension, intrauterine growth restriction, and oligohydramnios, are affected by women's choices both before and during pregnancy.

#### Sampling and recruitment for Phases 2 and 3

A total of 20-24 women (10-12 Aboriginal and immigrant) will participate. A Clinical Nurse Specialist at the LHHW has explored the potential response to this study and received positive feedback from women of various ethnocultural backgrounds attending the Perinatal Clinics. This sample size is based on the concept of data saturation--when further interviews do not elicit new information. Past research has indicated that this sample size frequently results in data saturation [54] although final sample size will be dependent on when saturation is achieved.

We will use purposive sampling. For Aboriginal women, we will recruit those who have largely lived in urban locales or reserve communities and women who live with or are separated from extended kin. The immigrant sample will consist of women from Sudan and China. These groups have been strategically chosen to represent two communities (in terms of country of origin) which are both relatively large in our study location and which reflect a substantial portion of the user profile at our selected maternity care unit. We will select immigrant participants with a range of migration experiences, such as length of time since entry to Canada (newcomers versus established immigrants) and social class trajectory post-migration to Canada. The inclusion criteria for newcomers will be two to four years of residence in Canada, and that for established immigrants more than five years. The range for newcomers is chosen because participants will have had some time to have become at least somewhat familiar with the Canadian health care system and hopefully will have some familiarity with English if this was limited prior to arrival in Canada. Nevertheless, language ability will not be an inclusion criterion, for translators will be hired and used for interviews. It is expected that regardless of fluency in English, some concepts do not translate well between languages, and may be best expressed in one's native tongue; thus, participants will be free to speak in whichever language they feel most comfortable.

The Maternal Fetal Medicine and Obstetric Medicine Clinics at the study site triage approximately 20 requests for referral daily. At the first clinic appointment, the Clinical Nurse Specialist (DG) or Nurse Educator (JF) will identify potential women who fit the inclusion criteria. A brief verbal description of the study will be provided to the women and an information letter will be offered should the woman appear to understand written English. The patient's potential willingness to participate will be assessed, and written consent will be obtained for contact by the study team. In addition to contact information, the consent for contact form includes a space where the woman can identify her preferred written and spoken language, such that the team can identify an interpreter to proceed with the contact, informed consent and study procedures. The team, with or without an interpreter as suitable, will then meet each interested women to gain written informed consent and continue with the study. The Aboriginal women will be recruited with the aid of the Aboriginal Cultural Helper. Care and caution will be exercised in respect of strict adherence to a federal guidance framework for research with Aboriginal peoples [55].

#### Methods

The camera as a research tool is well documented in disciplines such as anthropology and sociology [56-58]. Highley and Ferentz [59] maintain that the process of photography often leads to uncovering misconceptions and arriving at more reality-based understandings of phenomena. Hagedorn [60] refers to photographs as a medium to capture visual data of experience just as audio taping records verbal descriptions of experience. However, using photography as the sole data in qualitative research is insufficient. Photographs, like any form of art, can be interpreted in many ways and methods employing them must be grounded in the interactive context in which they acquire meaning [58]. Thus a combination of photographs and accompanying narratives adds richness to data in qualitative studies. ***Photovoice ***is the process by which people identify, represent, and enhance their community through specific photographic technique [61]. The technique entrusts cameras into the hands of community members, with the acknowledgement that their perspectives are valuable and necessary to the understanding of a problem or event [62]. Through small or large group discussion, community members reflect on the images produced in a safe environment. Therefore, as a data collection method, photovoice serves the dual process of engaging communities on a topic of concern while providing valuable information about their current life in relation to a topic. This approach is particularly useful for individuals who speak English as an additional language. Photovoice has been used in studies with ethnically-diverse groups including African American breast cancer survivors [63] and in a study of politics of representation in Guatemala and South Africa [64]. Founded on literature in education for critical consciousness, feminist theory, and documentary photography [65] the power of photovoice lies in its use of visual image as a form of communication, while the process of taking photos and discussing them with others serves to empower participants [62]. Using individual interviews focusing on the photographs (***photo-assisted interviews***) can also be incorporated, as for this research.

#### Data collection

**Figure 1:**
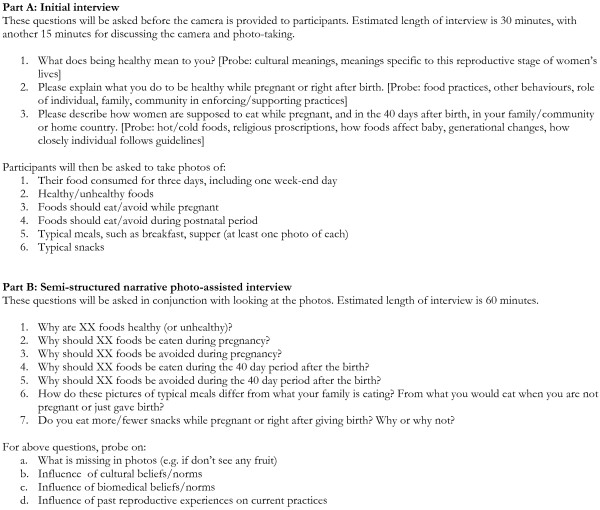
**Interview guides for initial interview (Part A) and follow-up photo-assisted interview (Part B)**.

After obtaining informed consent, a short initial interview (30 mins) will be undertaken and then disposable cameras will be provided to participants (the women will receive training on their use) and then the women will be asked to take photographs of all meals and snacks (including drinks) during a three-day period (including one weekend day) and other foods they perceive to be healthy/unhealthy for consumption during and after pregnancy. The women will be asked to hand in their camera to the Clinical Nurse Specialist, who will hand them over to the academic research team members (GH and HV) for development. Subsequently, a semi-structured narrative photo-assisted interview will be conducted (GH, HV or research assistant), where each woman will be asked to tell their story through the photos, to discuss whether food choices represented are typical or not, what factors influences their dietary choices, and what they would like to change. Figure 1 contains the interviews guides for both the initial and photo-assisted interviews. This will reveal not just what women are typically eating but the kinds of everyday issues that influence their food practices. Some questions will address culturally normative practices surrounding maternal food choices and consumption, including how women negotiate normative practices within their own worldview and experiences. A methodological approach of photovoice known as photo-elicitation, will also be used to complement interviews and better attain an understanding of taken-for-granted beliefs and assumptions about food practices during pregnancy and the postnatal period. Total time commitment for the participants is anticipated to be about 4 hours; they will be given a small honorarium in appreciation.

#### Data management and analysis

Data will be stored, managed, classified and ordered with the aid of Atlas.ti qualitative data analysis software (ATLAS.ti Scientific Software Development GmbH, Germany) with which the investigators have experience. Atlas.ti is useful for this study because it facilitates analysis of visual representations. The process of analysis is characterized by identification and classification of data and progresses to abstract generalizations and explaining patterns. We will draw upon the work of Miles and Huberman [66] which identifies eleven processes for analysis: 1) familiarization with the transcript, 2) identification of open and *in vivo *codes, 3) utilization of both theoretical and commentary memos, 4) funnelling and rationalization of redundant codes, 5) creation of themes, categories and families, 6) creation of graphic network views in Atlas.ti demonstrating relationships between the various codes and categories, 7) constant interrogation of the data and the challenging of initial assumptions, 8) identification of outliers and non-confirming data, 9) reflective team meetings to achieve higher level of abstraction in the analysis, 10) creation of hierarchies, classifications and typologies, and 11) creation of a written narrative. This characterizes the iterative process associated with qualitative analysis as preliminary interpretations are challenged and data are revisited in the light of further data collections and new insights into the data. Our goal is to develop a successful approach to perinatal nutrition counseling that is grounded in the experiential knowledge of immigrant and Aboriginal women. The study will be informed by a postcolonial approach to research, underlining the intersectionality of ethnicity, gender, and class, and how contemporary migrations reflect a historical legacy of power and colonialism [67]. The diversities of experiences are paramount, and therefore a critical lens will be applied to the categories used in our analyses and representations [68].

#### Study compliance and retention

Although we have in the past received excellent commitment from participants whenever photovoice methodology was used, we do realize the challenge of retaining participants between the first and second meetings during phases 2 and 3. The main strategy to overcome this issue will be the provision of a cash ($50) honorarium at the commencement of the second meeting when the photo-assisted interview is conducted. The information letter provided during the informed consent procedure will clearly explicate this procedure. Given that regular attendance at the study locations is already encouraged for medical reasons during the perinatal and especially prenatal periods, attendance by participants at the study meetings is expected to be very good.

### Phase 4 - Production of Culturally-Appropriate Visual Educational Tools

Using the findings from Phases 1 through 3 and after consultation with maternity nurses and allied health professionals (primarily dietitians), we will produce a culturally appropriate visual tool for each of the three ethnocultural groups, related to food choices during the perinatal period, for use in the perinatal clinic setting and other clinical areas in the LHHW. The first author (GH) was involved in the development of a similar tool in the United Kingdom, designed to enable non-English speaking women to express their feelings in the post-natal period http://www.cairnsbookshop.co.uk/search?q_filter=title&q=How+are+you+Feeling&submit. With permission, the photographs taken by the participating women will be used where suitable in order to capture real life examples of healthy or otherwise food choices.

#### Ethical Considerations

Approval has been obtained for this study from the Institutional Ethics Review Board for the University and the provincial health authority (Alberta Health Services). Voluntary written informed consent will be obtained from all participants. As part of the consenting process, participants will be assured that they do not have to answer every question, can choose to be audiorecorded (or not), and can withdraw at any time. To ensure that participants are fully informed, a translator will be used for both the consenting and interviewing processes. The principles of informed consent, confidentiality and anonymity will be observed at all times (including storage of materials). If instances of extreme distress (e.g. culture shock) are encountered, participants will be offered information about immigrant or other applicable support agencies. The interviews will be performed in a private room at the maternity unit, or at the women's house should she prefer this location.

#### Publication/Dissemination Plan - Integration, Synthesis and Knowledge Translation

The Canadian Institute of Health Research's (CIHR) knowledge translation (KT) framework and guidance documents [69,70] and Guidelines for Health Research Involving Aboriginal People [71] will guide the research team's KT strategies. CIHR defines KT as "a dynamic and iterative process that includes synthesis, dissemination, exchange, and ethically-sound application of knowledge to improve the health of Canadians, provide more effective health services and products and strengthen the health care system" [69] KT within this framework involves an active exchange of information between the researchers who create new knowledge and those who use the knowledge. Furthermore, KT is integrated in all steps between the creation of new knowledge and its application at the end of the grant to yield beneficial outcomes for society. Our goal is to create change within local policy and practice to gain a measurable and direct impact on health care for women using maternity care services who speak English as an additional language. In addition to our target audience of maternity nurses, and dieticians and managers at the LHHW, the results will be of use to other health care providers, policy makers, community groups, and immigrant-serving agencies. As such, a multi-level integrated KT and dissemination plan is anticipated to ensure key messages are delivered to various stakeholders.

#### Goals

The KT goals for this project include: A) the implementation of the research findings to nursing practice in respect of existing health service delivery in maternity care, B) engagement of key decision makers at the RAH/LHHW in respect of local policy changes that support the implementation of the findings, C) transferability of novel approaches in health promotion for immigrant and Aboriginal patients and families to other clinical specialities, and D) identification of new research opportunities and funding streams. It is anticipated that this research will provide excellent preliminary evidence to support a national study with one or more of the ethnocultural groups under consideration.

#### KT audience

The principal audience for the KT will be maternity care nurses (Registered Nurses, Licensed Practical Nurses, Nursing Aides) who deliver care including patient education to immigrant and Aboriginal women at the RAH/LHHW. The target audience will also include clinical educators, nurse managers and dietitians. This is a collaborative study therefore clinical nurses (DG, JF) have been involved from conceptualization and design of the study and will be involved through to dissemination. The KT dimensions are also an important component of research capacity building.

#### KT strategies

Consultation has taken place with clinical investigators at the conceptualization stage regarding the best approaches for engagement. We intend to deliver the findings of our study at the study site's research event in addition to the wider clinical and research community. The outputs (graphic/pictorial tools) from Phase 4 may have applications in other clinical specialities. We will produce a final report and a one-page research briefing paper, both of which will be written in plain English, freely accessible, and disseminated widely depending on the audience. We will publish in open access journals to enable nurses and other clinical staff to access our research findings readily.

## Discussion

The findings of this study will provide practitioners with an understanding of the cultural differences that affect women's dietary choices. We expect that the developed resources will be of immediate use to nurses, allied health staff, and other practitioners within the women's units, and will enhance their counseling efforts. The dissemination of the information into the wider community may have a greater long term impact in the primary and secondary prevention of these high risk conditions.

Our major concern will be the cultural safety of all participants - that is the recognition of power relationships between researcher and participants, the historical antecedents that have created these power relationships, and the promotion of well-being [72-74]. Hence, this research will recognize the processes of colonization that Aboriginal women have experienced in Canada, and that many immigrant women may have experienced in their home countries, and we will acknowledge the primacy of white Eurocentric perspectives in the Canadian context. The concept has been widely embraced by Aboriginal groups in Canada, but the key tenets and axioms of cultural safety are equally pertinent to observe in research with immigrant populations. To ensure the cultural safety of participants at all study sites, we will also adhere to the principles for research with immigrant groups established by the first author (GH) in collaboration with international colleagues [75,76]. Of importance will be concepts related to (a) mediating between the need to "fix" ethnic categories at various points in the research cycle, while still creating space for understanding the fluid and contingent nature of individual and collective identities; and (b) providing meaningful opportunities for marginalized people to co-produce policy-relevant findings, and recognizing the resulting implications for what we consider as "evidence."

## Competing interests

The authors declare that they have no competing interests.

## Authors' contributions

GH and HV conceptualized the study and prepared the draft of the research proposal. DG, JF, RM and FM provided comment on the proposal and DG solicited comment and feedback from potential study populations of their willingness to participate in this type of research. All authors assisted with manuscript preparation and approve of the final submission.

## Pre-publication history

The pre-publication history for this paper can be accessed here:

http://www.biomedcentral.com/1471-2393/11/100/prepub
